# The (Hour)glass Half-Full: Modified Silicone Hourglass Stents for the Treatment of Central Airway Obstruction

**DOI:** 10.7759/cureus.15501

**Published:** 2021-06-07

**Authors:** Bruce Sabath, Roberto F Casal

**Affiliations:** 1 Pulmonary Medicine, University of Texas MD Anderson Cancer Center, Houston, USA

**Keywords:** stent, silicone stent, rigid and fiber-optic bronchoscopy, lung cancer, airway obstruction

## Abstract

Central airway obstruction often presents with airway narrowing of differing internal diameters. Conventional straight stents do not fit these airways well and are prone to migration. We present a series of cases where hourglass-shaped silicone stents were customized intra-operatively to fit airway obstructions of both malignant and non-malignant etiologies and to improve patient performance status. Modified hourglass stents are a versatile tool to manage inoperable airway obstruction with unique anatomical characteristics.

## Introduction

Central airway obstruction is a commonly encountered problem in thoracic medicine. It is associated with a variety of pathologies, including malignancy and inflammatory conditions such as post-intubation and post-tracheostomy tracheal stenosis [1]. Notably, it has been suggested that the coronavirus pandemic will result in a widespread incidence of airway obstruction due to the high number of patients requiring prolonged mechanical ventilation, and such cases are already being reported [2-4].

Silicone stents have been used in the treatment of central airway obstruction for decades and are a mainstay of the standard of care [1]. They are commercially available as Y-shaped stents (to simultaneously stent trachea and mainstem bronchi) and also tubular stents (for tracheal or bronchial stenosis). Of note, each limb of the Y-stent or tubular stent has a fixed diameter. However, in many cases, central airway obstruction takes a more complex shape, with airways that abruptly change diameter, and consequently will not allow single-diameter stents to fit properly.

To rectify this issue, a variation of the silicone stent, i.e., the hourglass stent, has been designed. As the name implies, hourglass stents come in the shape of an hourglass, with the center diameter (the “waist”) smaller than the two ends. This design was created to prevent stent migration, with the narrow portion of the stent centered at the site of obstruction, and the larger portions proximal and distal to the obstruction.

However, we have found that hourglass stents can be made even more versatile. We have recently described a case where we created a miniaturized Y-stent out of an hourglass stent. A conventional hourglass stent was divided along its length to create two small limbs that fit into stenotic postsurgical airways, which would have been very difficult to treat otherwise [5]. Hourglass stents can be tailored and used in other ways and for various etiologies. Through this small series, we illustrate how hourglass stents can be customized to restore patency to airways with uneven luminal diameters. To our knowledge, this is the first report on this topic in the medical literature.

## Case presentation

Case 1

**Figure 1:**
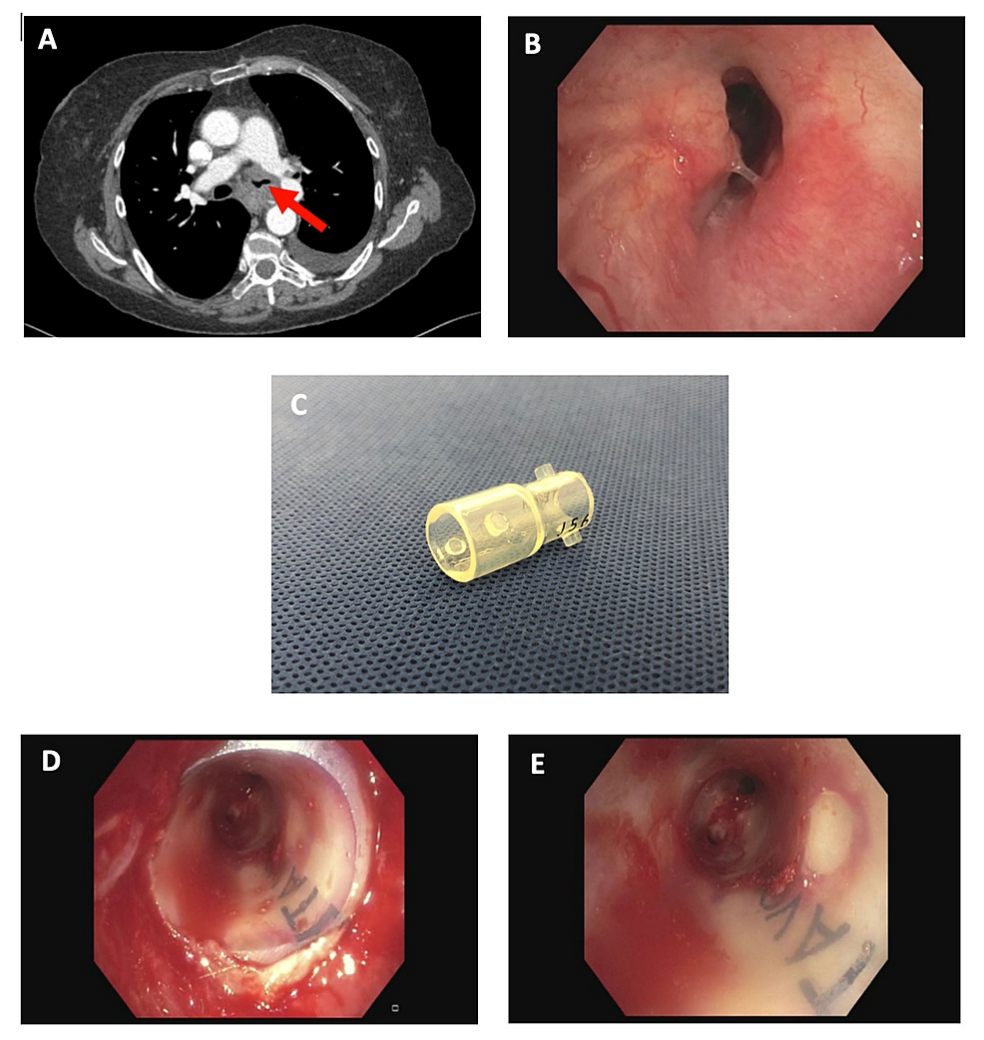
Left mainstem bronchial obstruction treated with modified hourglass stent A: CT demonstrating LMSB obstruction (arrow). B: Bronchoscopic image of LMSB obstruction. C: Modified hourglass stent. D: Post-stent insertion into the LMSB, proximal end. E: Post-stent insertion into the LMSB, distal end CT: computed tomography; LMSB: left mainstem bronchus

A 65-year-old woman with metastatic lung adenocarcinoma presented to our interventional pulmonology clinic complaining of worsening dyspnea for over two months. CT imaging noted narrowing of the left mainstem bronchus (LMSB) due to an enlarging subcarinal lymph node (Figure 1A). Bronchoscopy showed the LMSB to be 70% narrowed due to focal extrinsic compression from the underlying node (Figure 1B). The remainder of the LMSB and the upper and lower lobes were uninvolved and fully patent. As there was no endobronchial tumor to debulk, a stent was indicated. Due to the uneven diameter of the airway, we felt that a self-expandable metal stent could easily migrate proximally. As such, we customized a 12 x 10 x 12-mm hourglass stent by dividing it in half across its smaller diameter (Figure 1C). This was inserted via a rigid bronchoscope into the LMSB with the 10-mm waist fitting across the obstruction and the 12-mm portion distal to the obstruction, opening to the left upper and lower lobes (Figures 1D, 1E). The airway was then fully patent. The patient tolerated the procedure well and immediately noted improved dyspnea in the recovery room. At the follow-up, she noted that her improvement was significant and she was now able to be much more active. After two months, it was found that systemic therapy had resulted in a significant reduction in overall tumor burden including at the LMSB, and the patient's stent was removed.

Case 2

**Figure 2:**
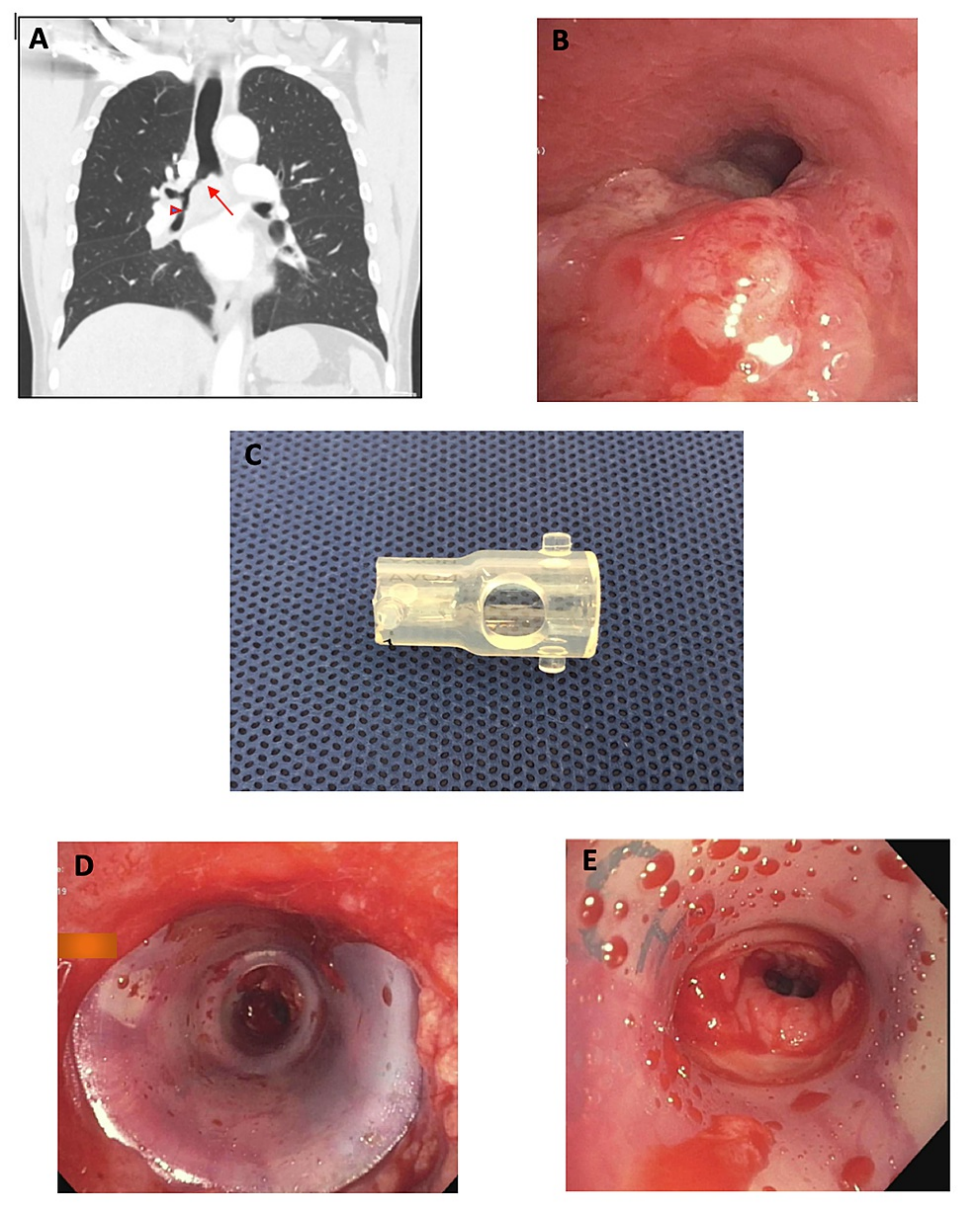
Right mainstem bronchial and right bronchus intermedius obstruction treated with modified hourglass stent A: CT demonstrating RMSB (arrow) and RBI obstruction (arrowhead). B: Bronchoscopic image of RMSB tumor causing significant narrowing of the airway. C: Modified hourglass stent cut in half with side-hole for right upper lobe bronchus. D: Inserted modified hourglass stent, with larger diameter in RMSB and smaller diameter in RBI (proximal view). E: Inserted modified hourglass stent, with larger diameter in RMSB and smaller diameter in RBI (distal view) CT: computed tomography; RMSB: right mainstem bronchus; RBI: right bronchus intermedius

A 57-year-old man presented to our emergency department with intermittent cough and hemoptysis. He had been diagnosed with non-small cell lung cancer in the preceding weeks but had not yet started treatment. Imaging noted a large right lower lobe mass with compressive atelectasis and narrowing of the right mainstem bronchus (RMSB) and right bronchus intermedius (RBI) (Figure 2A). Upon bronchoscopy, a mixed (intrinsic and extrinsic) 70% obstruction of the RMSB, RBI, and right upper lobe was found (Figure 2B). The endobronchial component of the obstruction could be debulked but an extrinsic obstruction remained in the RBI, which necessitated stenting. Given the smaller diameter of the bronchus intermedius with respect to the RMSB, a customized hourglass stent (12 x 10 x 12 mm) was selected to match the narrowing. One of the wider ends of the stent was cut off and a window was then created on the remaining wider portion of the stent to allow aeration of the right upper lobe (Figure 2C). This stent was inserted via rigid bronchoscopy with the wider end proximally to match the larger RMSB and the stent window facing the right upper lobe take-off (Figures 2D, 2E). The patient tolerated the procedure well and an improvement in dyspnea was noted thereafter.

Case 3

**Figure 3:**
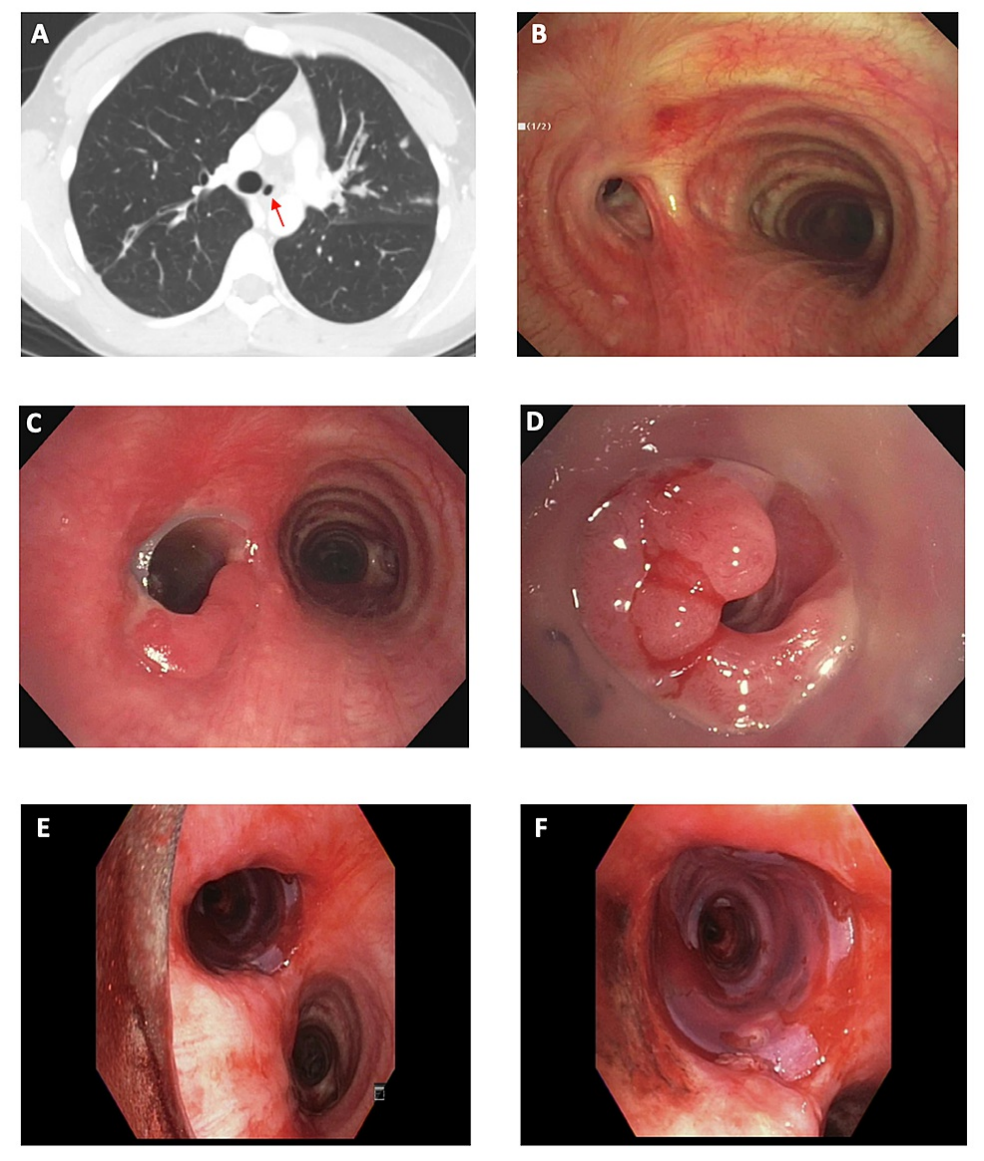
Severe left mainstem bronchial stenosis due to tuberculosis treated with modified hourglass stent A, B: CT and bronchoscopic images showing severe LMSB stenosis (arrow). C, D: Significant granulation tissue complicating straight silicone stent. E, F: Modified hourglass stent fitting well in the LMSB CT: computed tomography; LMSB: left mainstem bronchus

A 26-year-old woman with a history of pulmonary tuberculosis presented to our clinic with dyspnea. She had completed nine months of directly observed therapy three years prior but had been experiencing dyspnea since then. For employment purposes, she underwent a chest X-ray, which noted a possible airway abnormality. The follow-up CT confirmed LMSB stenosis (Figure 3A). Surgical consultation recommended left pneumonectomy but she declined.

Bronchoscopy revealed a significant fibrotic stricture of the LMSB (Figure 3B). This was recanalized using an electrocautery knife and balloon dilation. However, within one week, she began to experience worsening dyspnea again. Repeat bronchoscopy noted the recurrence of LMSB stenosis, and a straight silicone stent was placed. This did not fit well, but attempts were made to modify its shape and it was left in place. With this intervention, her symptoms improved temporarily, but she required two more bronchoscopies over the ensuing four months for recurring symptoms; stent-related granulation tissue had resulted in an uneven airway with mild obstruction proximal to the stent but significant distal obstruction (Figures 3C, 3D). Ultimately, during repeat bronchoscopy, the stent was removed as per the patient's request and because of a suspected stent infection. However, the entire LMSB collapsed immediately due to significant bronchomalacia, requiring a new stent. At this point, given the conical shape of the airway, an hourglass stent was modified to match her airway. One end of a 14 x 12 x 14-mm stent was cut and the stent was placed with the wider end in the proximal LMSB. This fit the anatomical variation of the airway and completely recanalized the LMSB (Figures 3E, 3F).

Thereafter, despite requiring four bronchoscopies over a period of eight months, the patient did well and required only one other bronchoscopy five months later to manage granulation tissue. The stent itself remained in place for 10 months. The patient was found to be doing well at the last follow-up.

## Discussion

Silicone stents have been used with great success for relieving central airway obstruction for decades [6]. While customization with regard to length or side openings of single-diameter stents is feasible, it will not solve the problem of airways that abruptly change diameter, such as the ones described in this series.

Customized hourglass stents can provide a simple and ready solution for this common problem as they allow for two diameters to be present within the stent. This allows for stenting of airways with an uneven diameter, whereas a stent with a single diameter is unlikely to fit properly. Standard stents are prone to migrate in obstructions such as these because the stent can only match the narrowest portion of the airway; the remainder of the stent must then be relatively loose in the portion of the airway with a larger diameter. Moreover, stents that do not fit well are subject to repetitive motion during breathing; this repetitive mucosal trauma has been associated with the development of granulation tissue, which may predispose to further obstruction and infection [7]. This phenomenon was demonstrated in our third case where the conventional stent did not fit the airway and was associated with exuberant granulation tissue requiring frequent bronchoscopies.

We have demonstrated that simple modifications to widely available hourglass stents can provide customizations to fit a patient’s anatomy and pathology - and can result in improvements in both malignant and non-malignant diseases. In our first case, a simple modification produced a stent that could completely open an airway and restored the performance status of the patient. Our second case required further modifications to the hourglass stent, including the creation of an opening for the right upper lobe, but this also reestablished aeration to a previously complex airway. In our third case, due to recurrent post-tuberculous bronchostenosis, a straight silicone stent did not match the airway, but only led to further complications. However, once a modified hourglass stent was used, the patient was able to go longer periods without requiring interventions. Of note, the stents used were all commercially available and composed of medical-grade silicone that met official biocompatibility requirements.

Finally, consideration must be given to how therapeutic bronchoscopy might advance further in the future. This pertains to the ability to produce stents that are personalized to an even greater level of detail than what we described in this series. Three-dimensional printing offers such potential and, while it is still in its early stages of development, it has already proven feasible in this regard. Three case reports describing five cases of airway obstruction treated with stents made with the aid of three-dimensional printing have been reported in the literature so far [8-10]. Ideally, such research and development will continue to advance and will one day make these advanced devices available at the point of care rather than through external manufacturers.

## Conclusions

Proceduralists should consider how they might innovate and modify what they already have in their armamentarium. Hourglass stents may be underutilized and, in our experience, offer an additional level of versatility over more commonly used Y and straight silicone stents.
